# OS and DFS are affected by different diagnostic methods and hysterectomy procedures in endometrial cancer patients: A single‐center retrospective study

**DOI:** 10.1002/cam4.6465

**Published:** 2023-08-16

**Authors:** Haiying Sun, Long Zhang, Peiying Fu, Ronghua Liu

**Affiliations:** ^1^ Department of Obstetrics and Gynecology, Tongji Hospital, Tongji Medical College Huazhong University of Science and Technology Wuhan People's Republic of China

**Keywords:** dilation and curettage, endometrial cancer, hysterectomy, survival analysis

## Abstract

**Purpose:**

We aimed to evaluate whether hysteroscopy increases the risk of intraperitoneal dissemination or worsens the prognosis of endometrial carcinoma (EC) patients and whether radical hysterectomy (RH) improves overall survival (OS) or disease‐free survival (DFS) in patients with stage II to III EC and to investigate the effects of different procedures for identifying EC and the effects of different surgical methods on the OS and DFS of endometrial cancer patients.

**Methods:**

Four hundred sixty‐five women with EC were included in this retrospective study. Log‐rank tests and Kaplan–Meier analysis were used for the outcome comparisons of the effects of the EC diagnostic method and different hysterectomy procedures. A Cox proportional hazards model was used for univariate regression analysis.

**Results:**

Among the three procedures for diagnosing EC (diagnostic curettage, hysteroscopy, and hysterectomy), the incidences of fallopian tube and ovarian invasion were not significantly different (*p* = 0.506 and 0.066, respectively). The diagnostic methods for EC had no significant effect on OS (*p* = 0.577) or DFS (*p* = 0.294). In addition, type II RH and type III RH did not improve the prognosis of patients with FIGO stage II and III disease (log‐rank *p* = 0.914 and 0.810 for OS; log‐rank *p* = 0.707 and 0.771 for DFS, respectively).

**Conclusion:**

Based on the current study evidence, the use of diagnostic hysteroscopy procedures is safe and does not increase the risk of fallopian tube and ovarian invasion of intraperitoneal dissemination or worsen the prognosis of EC patients. Type II and type III RH did not demonstrate a benefit for stage II‐III EC patients.

## 1 INTRODUCTION

Endometrial carcinoma (EC) is the second most prevalent gynecological malignancy in the female reproductive tract, with an increasing incidence in China according to the National Central Cancer Registry of China (NCCR). Both the incidence of EC and the EC‐associated mortality rate are increasing with improvements in living standards and changes in lifestyle.^1^ Many studies have revealed that the 5‐year overall survival (OS) of early EC patients is as high as 90%, but the corresponding rates for stage III and IV EC patients are only 57%–66% and 20%–26%, respectively.^2^ Accumulating evidence suggests that the management of EC should be personalized taking into account the performance status of the patient, in particular in case of elderly women.^3^ Thus, it is necessary to detect, diagnose and treat EC early in clinical practice.

Diagnostic curettage [i.e., dilation and curettage (D&C)] is the most commonly used procedure. However, D&C is associated with incomplete curettage and unforeseen limitations. Hysteroscopic biopsy can address the above limitations, reducing the rate of missed diagnosis and the risk of uterine adhesions. In addition, hysteroscopy enables comprehensive observations of tumor extent and morphology or cervical canal invasion to preliminarily judge the tumor stage. One research^4^ has shown that the diagnostic rate of hysteroscopy for EC is relatively high, with approximately 89% accuracy. However, because hysteroscopy involves increasing intrauterine pressure with distension media, it may be associated with the increased potential for cell dissemination into the peritoneal cavity, causing upstaging and potentially affecting prognosis. In their meta‐analysis, Polyzos et al found that hysteroscopy increased the risk of cancer cell dissemination.^5^ Furthermore, in their cohort study, Chen et al found that diagnostic hysteroscopy may increase the rate of positive peritoneal cytology at the time of surgical staging.^6^ In another study, compared with the control treatment (endometrial biopsy or D&C), hysteroscopy resulted in a significantly higher positive peritoneal cytology rate (OR, 1.82; *p* = 0.0004), and the resulting recommendation was to maintain the medium inflation pressure below 80 mmHg.^7^ However, there is more evidence suggesting that hysteroscopy may not increase the risk of intraperitoneal dissemination of EC cells or affect the survival of women with this disease. For example, one retrospective cohort study found that diagnostic hysteroscopy had no adverse effects on the incidence of positive peritoneal cytology or on prognosis in stage I EC patients.^8^ Consistently, other researchers observed this lack of effect on the FIGO stage or prognosis of EC patients after prolonging the time interval between D&C and hysterectomy.^9^ Thus, it is necessary to explore the relationship between hysteroscopic procedures and poor prognostic outcomes.

We are also interested in the impact of different surgical strategies on the prognosis of patients with EC. Current guidelines for the ideal management of endocervical stromal invasion or more advanced EC are currently being debated. There are three main surgical procedures available: extrafascial hysterectomy (EH), type II radical hysterectomy (RH), and type III RH. The most challenging part of the RH procedure is parametrectomy. Histologically, parametrial involvement has been demonstrated in 11.5% of patients with stage II EC and in 52.9% of patients with stage III disease.^10^ It has been hypothesized that RH may remove parametrial metastasis in EC patients with cervical involvement.^11^ Some studies revealed that the prognosis of patients who underwent type III RH was better than that of patients who underwent EH.^12^, ^13^ However, RH surgery for stage II EC is associated with obvious perioperative complications, including blood loss, vessel injury, nerve injury and dysfunction, ureteral injury, voiding dysfunction, prolonged hospital stay, lymphocyst formation, and fistula, which also significantly affect the quality of life (QoL) of EC patients and result in complex postoperative complications. Nonetheless, different points of view are presented in the literature, namely there is no intergroup difference between EH and RH, and type III RH can increase intraoperative blood loss and perioperative complications.^14^ In multiple studies, a lack of benefit with RH was demonstrated, even when postoperative adjuvant radiotherapy therapy was added. Therefore, it is controversial whether these patients benefit from RH.

To address the above controversies, this study aimed to investigate whether the EC detection methods and different hysterectomy procedures affect the disease‐free survival (DFS) and OS of patients with EC. This information will allow clinicians to understand the significance of hysteroscopy in the diagnosis of EC more clearly and choose the best surgical method.

## 2 MATERIALS AND METHODS

### 2.1 Patients

Clinical data were obtained from medical records from 2006 to 2016 in our database and retrospectively reviewed. After exclusions, a total of 465 patients fulfilled the inclusion criteria, and their data were reviewed. The following clinical data were retrieved: age at diagnosis, height and weight, stage of disease, methods used to find the EC, histologic subtype, surgical protocol, and follow‐up data. All protocols were approved by the Ethics Committee of Tongji Hospital, Tongji Medical College, Huazhong University of Science and Technology. All eligible patients provided informed written consent before entering this study.

### 2.2 Inclusion criteria

All patients were enrolled based on the following criteria: had endometrial cancer that was pathologically confirmed by two pathologists; were aged <80 years; underwent surgery for hysterectomy at Tongji Hospital.

### 2.3 Exclusion criteria

The exclusion criteria were a Kanefsky performance status <80, age greater than or equal to 80 years, and previous history of cancer. The EC patients who accepted hysterectomy surgery in other hospitals were excluded. Patients with active infectious disease requiring systemic treatment or other medical complications and women who lacked information on clinical risk factors were also excluded from this retrospective study.

### 2.4 Diagnosis or surgical methods

#### 2.4.1 Hysteroscopy

All patients underwent ultrasound or magnetic resonance imaging (MRI) before hysteroscopy or D&C. Most patient specimens were obtained during blind fractional D&C. Endocervical curettage was performed before the hysteroscopic procedure. This hysteroscopic procedure was performed under intravenous anesthesia using saline solution as a distension medium adjusted to a pressure lower than 100 mmHg. Endometrial specimens were obtained with biopsy forceps, flexible scissors, or bipolar electrode resection loops. Our hysteroscopic surgeries were performed by experienced doctors, reducing the duration of surgery, and normal saline was used as the distention medium (room temperature).

#### 2.4.2 Hysterectomy procedures

All surgeries were performed by trained gynecologic oncologists. Peritoneal cytology was performed for patients who underwent peritoneal washings. Patients underwent a surgical clinical staging procedure consisting of different kinds of hysterectomy, bilateral salpingo‐oophorectomy, bilateral pelvic lymphadenectomy and/or para‐aortic lymph node dissection. The Piver–Rutledge classification was used to describe the procedure of hysterectomy.^15^

#### 2.4.3 Extrafascial hysterectomy

EH involved complete removal of the uterus, cervix, and a small rim of the vaginal wall at the fornix level. This technique kept the paracervical ring intact, and the paracolpos was removed.

#### 2.4.4 Type II radical hysterectomy

The anterior layer of the vesicouterine ligament was resected. The uterus and an extra 1.5–2‐cm cuff of the vaginal wall were removed, and a wide local excision was made in the parametrial and paravaginal tissues. Additionally, in this procedure, more of the cardinal ligament was resected than in EH.

#### 2.4.5 Type III radical hysterectomy

The uterine artery was ligated at its origin at the internal iliac artery. The uterus and upper third of the vaginal wall were removed. The paravaginal and parametrial tissues were widely excised near the pelvic wall.

All specimens were reviewed by the same two gynecologic pathologists. Classification by histological type, grade, fallopian tube invasion, ovarian invasion, and stage was based on criteria adopted by FIGO for EC. Patients received postoperative adjuvant chemotherapy and/or radiation therapy based on their pathological risk factors.

### 2.5 Follow‐up

The follow‐up assessments were designed to be conducted every 3 months in the first year and every 6 months in the next 4 years after treatment during office visits. According to our database, for a proportion of patients, follow‐up was not performed due to loss of contact, and the data from these cases were excluded from the survival analysis. The OS and DFS rates were calculated from the day of diagnosis until the date of first relapse or death, respectively.

### 2.6 Statistical analysis

Log‐rank tests and Kaplan–Meier analysis were used for the DFS and OS comparisons of the effect of the EC diagnosis method and different hysterectomy procedures. Chi‐squared tests were used to identify the factors that affected the method used to diagnose EC. A Cox proportional hazards model was used for univariate regression analysis to verify whether the methods for finding EC and surgery protocol predicted DFS. The median follow‐up time was calculated as the median observation time among all patients. IBM SPSS 26.0 statistical software was used to perform the statistical analysis. All reported *p‐*values were two‐sided, and we considered *p* < 0.05 to be the significance threshold.

## 3 RESULTS

### 3.1 Patient characteristics

In the study, we included 465 patients with stage I‐IV EC who received a hysterectomy at Tongji Hospital (Table 1). The mean age of the patients was 53.3 (25–76) years. The mean body mass index (BMI) was 23.8 kg/m^2^ (16.8r–36.5). Of the 465 EC patients, only 3 (0.3%) had a history of smoking. In total, 114 (26.1%) and 34 (7.9%) patients had a history of hypertension and diabetes, respectively. The most common histologic subtype was endometrioid adenocarcinoma, which was present in 74.8% (348) of patients. Endometrial cancer is often diagnosed by diagnostic curettage. In this study, diagnostic curettage, hysteroscopy, and surgery were used to diagnose EC in 74.9% (287), 2.4% (11), and 15.6% (73) of patients, respectively. Most of the histologic grades were G1 and G2 (68.4%). The proportion of fallopian tube and ovarian metastases was relatively low (4.5% and 5.6%, respectively). There were 324, 61 and 62 EC patients who underwent EH, type II RH, and type III RH, respectively.

**TABLE 1:** Characteristics of the endometrial cancer patients and tumors studied.

Variables	Value
Total (*N*)	465
Age, mean (range)	53.3 (25–76)
BMI, mean (range)	23.8 (16.8–36.5)
Smoking, *N* (%)	3(0.7%)
Hypertension, *N* (%)	114 (26.1%)
Diabetes, *N* (%)	34 (7.9%)
Ways of finding EC, *N* (%)	
Diagnostic curettage	350 (74.9%)
Hysteroscopy	11 (2.4%)
Hysterectomy	73 (15.6%)
Unknown	33 (7.1%)
Stage (FIGO)	
I	287 (61.5%)
II	39 (8.4%)
III	49 (10.5%)
IV	12 (2.6%)
Unknown	80 (17.1%)
Histologic subtype, *N* (%)	
Endometrioid	348 (74.8%)
Others	117 (25.2%)
Histological grade of endometrioid tumors	
Grade 1	179 (38.5%)
Grade 2	139 (29.9%)
Grade 3	41 (8.8%)
Others	106 (22.8%)
Fallopian tube invasion	
Yes	21 (4.5%)
No	418 (90.5%)
Unknown	23 (4.9%)
Ovarian invasion	
Yes	26 (5.6%)
No	423 (89.5%)
Unknown	23 (4.9%)
Surgical protocol	
Extrafascial hysterectomy	324 (72.5%)
Type II radical hysterectomy	61 (13.1%)
Type III radical hysterectomy	62 (13.3%)
Unknown	20 (4.3%)

### 3.2 Association of tumor characteristics with EC diagnosis

Diagnostic curettage was the most common method used to diagnose endometrial cancer (73.5%). FIGO stage I disease (79.2%, 55.6%, 61%) was the most commonly diagnosed stage for all three methods. There were significant differences between the three methods based on FIGO stage, with a *p*‐value of 0.015 (Table 2). Highly and moderately differentiated disease was the most commonly found by the three methods (91.4%, 100%, 82.7%), with no obvious differences (*p* = 0.398). The three methods differed in their diagnosis rates for fallopian tube invasion and ovarian invasion (*p =* 0.506 and 0.066, respectively), but there were no significant differences between the three methods in diagnosing EC. Interestingly, no patients who underwent hysteroscopy had fallopian tube or ovarian invasion (0/11, 0/11).

**TABLE 2:** Association of each EC diagnostic method with tumor characteristics.

Variables	Diagnostic curettage *N* (342)	Hysteroscopy *N* (11)	Hysterectomy *N* (63)	*p*
Stage (FIGO)				
I	235 (79.1%)	5 (55.6%)	36 (61.0%)	0.015
II	22 (7.4%)	3 (33.3%)	8 (13.6%)	
III	33 (11.1%)	1 (11.1%)	13 (22.0%)	
IV	7 (2.4%)	0 (0.0%)	2 (3.4%)	
Histological grade				
Grade 1	147 (52.9%)	5 (71.4%)	22 (42.3%)	0.398
Grade 2	107 (38.5%)	2 (28.6%)	21 (40.4%)	
Grade 3	24 (8.6%)	0 (0.0%)	9 (17.3%)	
Fallopian tube invasion				
Yes	13 (3.8%)	0 (0.0%)	4 (6.3%)	0.506
No	329 (96.2%)	11 (100%)	59 (93.7%)	
Ovarian invasion				
Yes	15 (4.4%)	0 (0.0%)	7 (11.1%)	0.066
No	327 (95.6%)	11 (100%)	56 (88.9%)	

### 3.3 Effect of EC diagnostic method on survival

**FIGURE 1:**
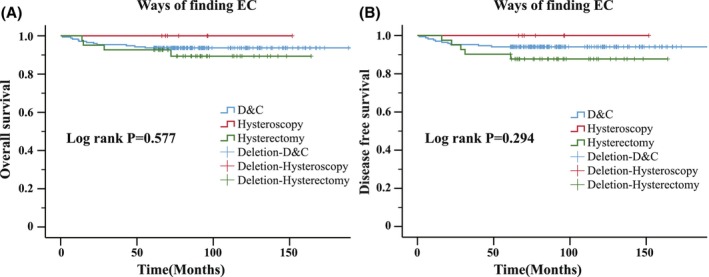
Kaplan–Meier survival curves according to diagnostic method. (A) Overall survival; (B) disease‐free survival.

A Cox proportional hazards model was used to investigate whether the diagnostic method for EC affected OS and DFS. In the Kaplan–Meier analysis, the method for diagnosing EC had no significant effect on OS (*p* = 0.577) or DFS (*p* = 0.294) (Figure 1). Due to significant differences between the EC diagnostic method and FIGO stage, we performed subgroup analyses. For patients with FIGO stage I EC, only two deaths occurred among those diagnosed with diagnostic curettage. For patients with FIGO stage II EC, three deaths occurred among those diagnosed with diagnostic curettage. For patients with FIGO stage III disease, seven deaths occurred. No deaths occurred among those with FIGO stage IV disease. The log‐rank *p*‐values of the effects of diagnostic curettage, hysteroscopy, and hysterectomy on OS in patients with EC were 0.812, 0.439, and 0.088, respectively. The results for DFS were similar to those for OS, with log‐rank *p*‐values of 0.731, 0.761, 0.607, and 0.480, respectively (Table 3).

**TABLE 3:** Cox analysis of the effect of each EC diagnostic method on survival based on stage.

FIGO stage	Method	OS			DFS		
		Total	Events	*p*	Total	Events	*p*
I	A	116	2	0.812	116	3	0.731
	B	3	0		3	0	
	C	21	0		21	0	
II	A	12	3	0.439	11	1	0.761
	B	1	0		1	0	
	C	5	0		5	0	
III	A	12	4	0.808	11	3	0.607
	B	1	0		1	0	
	C	8	3		8	4	
IV	A	3	0	–	2	1	0.48
	B	0	0		0	0	
	C	1	0		3	1	

Abbreviations: A, diagnostic curettage; B, hysteroscopy; C, hysterectomy.

### 3.4 Endometrial cancer outcomes are differentially affected by hysterectomy procedures

**FIGURE 2:**
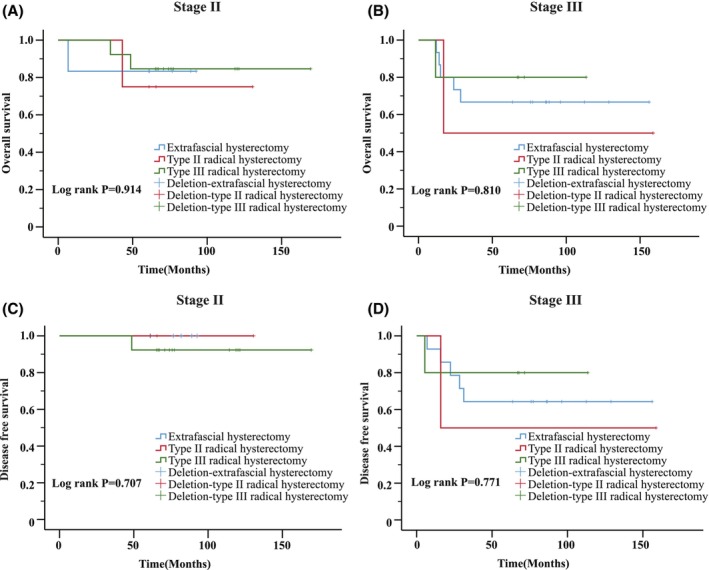
Kaplan–Meier survival curves according to the hysterectomy procedure used for patients with FIGO stage II EC (A. overall survival, log‐rank *p* = 0.914; C. disease‐free survival, log‐rank *p* = 0.707) and FIGO stage II EC (B. overall survival, log‐rank *p* = 0.810; D. disease‐free survival, log‐rank *p* = 0.771).

Because of the controversy regarding whether patients benefit from RH, an analysis was performed to compare OS and DFS rates using the Kaplan–Meier method among the groups with FIGO stage II and III disease who underwent different surgical protocols (EH, type II RH and type III RH). Among those with FIGO stage II disease, one, one, and two deaths occurred in the hysteroscopy, D&C, or hysterectomy groups, respectively, and among those with FIGO stage III disease, five, one, and one death occurred in the hysteroscopy, D&C or hysterectomy groups, respectively. Only one patient with FIGO stage II disease experienced tumor recurrence, and seven patients with FIGO stage III disease experienced tumor recurrence. We found that the log‐rank *p*‐values for the effects on OS and DFS were 0.914 and 0.707 among FIGO stage II patients (Figure 2). Similarly, there were no benefits for patients with FIGO stage III EC who underwent type II RH or type III RH compared with patients who underwent EH (*p* = 0.810 and 0.771 for OS and DFS, Figure 2).

## 4 DISCUSSION

With the increasing use of hysteroscopy, an increasing number of clinical doctors have raised concerns regarding its oncological safety. One study intended to identify whether diagnostic hysteroscopy increases the risk of intraperitoneal cancer cell dissemination, and 9 trials were included in their analysis (1015 patients). Hysteroscopy resulted in a significantly higher rate of positive peritoneal cytology and an obviously higher risk of disease upstaging owing to the presence of cancer cells compared with no hysteroscopy.^6^ In a retrospective study, there was a markedly higher rate of positive peritoneal washings after hysteroscopy compared to D&C but not an increased risk of adnexal, abdominal, or retroperitoneal lymph node metastases in EC patients.^16^ The same research team reached slightly different conclusions in 2016, and this phenomenon was observed only in patients with stage FIGO I endometrial cancer.^17^ Whether hysteroscopy increases the risk of intraperitoneal dissemination or worsens the prognosis of EC patients and whether RH improves OS or DFS among stage II to III EC patients is controversial. In our study including 465 EC patients, the incidences of fallopian tube and ovarian invasion were not significantly different (*p* = 0.506 and 0.066, respectively), and there was no significant difference in OS or DFS.

The main limitations of our study are the retrospective study design and the lack of some data, such as peritoneal flushing fluid cytology findings. We could analyze whether hysteroscopy affected the survival of patients with EC only from the follow‐up data, which makes it difficult to control for confounders such as adjuvant treatment with chemotherapy and radiation for advanced‐stage disease when evaluating the route of hysterectomy. There was some selection bias with patients undergoing hysteroscopy versus D&C, missed clinical characteristics (e.g., lymphovascular space invasion [LVSI]), and the high rate of loss to clinical follow‐up, which may have some adverse effects on the results of our study. Another limitation is that the number of stage II‐IV EC patients included was small. In addition, due to the long patient enrollment period, there are certain differences in the classification criteria of endometrial cancer, which will have unavoidable adverse effects on the grouping of patients. These data need to be further investigated. Despite these limitations, we feel that our study provides more information on the effect of HSC on peritoneal cytology and outcomes in type II EC.

Many researchers have reported positive peritoneal cytology rates, whereas others have not confirmed such changes. Hysteroscopy can improve diagnostic accuracy and, compared with D&C, did not increase the risk of intraperitoneal dissemination in 147 EC patients in a previous study.^18^ Some retrospective studies have shown that hysteroscopy with morcellation is a safe diagnostic procedure for EC, even high‐risk and type II EC, and that it does not lead to increased dissemination of cancer cells, LVSI or surgical upstaging of tumors.^19^, ^20^ A retrospective cohort analysis evaluated the outcome measures of 392 EC patients diagnosed by endometrial biopsy, D&C or hysteroscopy and found that hysteroscopy did not significantly affect the survival outcomes and recurrence of endometrial cancer.^21^ Two multicenter retrospective cohort studies evaluated 429 patients with stage II EC and 1324 EC patients. There were no differences between the 5‐year DFS rates and 5‐year OS rates of the diagnostic hysteroscopy group and that of the D&C group.^22^ Moreover, a recent study published by Quintana‐Bertó R et al. compared relapse and survival rates in 1731 women who had been evaluated by either pipelle biopsy or hysteroscopy in a retrospective multicentric study and found no impact on oncological outcomes.^23^ The present study reports agree with these findings.

However, some researchers have put forward different views. In particular, hysteroscopy does not improve the sensitivity of detection of endometrial hyperplasia or carcinoma.^24^ Additionally, the prognostic effect of hysteroscopy has not been well described in the literature for type II EC. To date, there have been no prospective studies comparing hysteroscopy to D&C in which a hysterectomy specimen serves as the gold standard to which both methods are compared. Nonetheless, our conclusions are consistent with those of the above literature. We compared three different methods of finding endometrial lesions, and there was no distinct difference in the impact of these three methods on patient prognosis in our study. We cannot compare the advantages and disadvantages of D&C and to those of hysteroscopy. Because this is a retrospective study, we could not perform comparisons of dilation fluid pressure in this study. Therefore, based on our own results and the conclusions presented in the literature, we cautiously suggest that hysteroscopy may be more conducive to finding endometrial lesions under the control of dilatation fluid pressure.

There are many options surgical approach for performing hysterectomy, including abdominal, robot‐assisted laparoscopic and vaginal natural orifice transluminal endoscopic surgery (vNOTES).^25^ It is still controversial whether RH affects endometrial cancer outcomes. RH with bilateral salpingo‐oophorectomy (BSO) is recommended for patients with stage II EC based on guidelines before 2014.^26^ However, since 2015, some studies have found that RH does not improve the prognosis of patients with stage II EC.^27^, ^28^ Nevertheless, the latest NCCN guidelines for uterine neoplasms in 2023 still recommend total hysterectomy or RH and BSO and surgical staging for stage II EC.^29^ The hypothesis was that RH could remove histologic parametrial involvement (PMI) in patients with cervical involvement because the incidence of PMI among FIGO stage II patients is 11.5%–14.1%.^10^ Therefore, the recommendations were that RH should be considered for patients with stage II EC. Some authors have emphasized the importance of parametrial dissection in managing these cases.^30^ Several retrospective studies have demonstrated the benefits of RH compared with SH in patients with stage II EC and showed that RH or modified RH might improve OS compared with SH, especially in women with more extensive cervical involvement.^31^ The type III RH in this series provided excellent local control and long‐term survival with minimal morbidity in patients with gross cervical involvement, and this method is recommended in this clinical area.^32^ However, no similar results were found in our study.

However, several clinical studies have reported contradictory results.^27^, ^33^ The type of hysterectomy was not identified as a prognostic factor in 180 EC patients; no survival benefits from RH were found and the procedure was more frequently associated with perioperative and urinary complications.^34^ Phelippeau et al. evaluated 7552 patients with stage II disease and found that the type of hysterectomy (RH) did not impact OS, even after adjusting for adjuvant radiation in a matched cohort study.^14^ Moreover, for high‐risk stage II EC, RH resulted in a shorter 5‐year OS and 5‐year cause‐specific survival than SH combined with vaginal brachytherapy (BT) in a retrospective cohort study. However, none of the patients in the RH group received vaginal BT, which may not be in line with clinical practice.^35^ A multicenter collaborative study reported that modified radical hysterectomy (mRH) did not reduce the rate of local recurrence compared with SH in 1335 EC patients, but surgical stages II and III did impact the rate. To date, the effect of mRH on progression‐free survival (PFS) and OS among stage 3 EC patients is unclear, and there have been no studies on this issue.^36^ There are some doubts about this conclusion. As mentioned by the authors, due to selection bias, more patients in the RH group underwent RH and/or radiotherapy. However, there was no difference in the percentage of postoperative adjuvant radiotherapy between the two groups of EC patients. This means that RH may play an important role in these patients.

## 5 CONCLUSION AND FUTURE DIRECTIONS

In this retrospective study, there were no differences in the survival outcomes of the endometrial cancer patients diagnosed by means of hysteroscopy, D&C, or hysterectomy. Compared with EH, RH and mRH did not improve OS and DFS among patients with stage II to III EC. We recommend the use of hysteroscopy with low‐distension medium pressure, which may be safe for EC patients. The benefits of RH were not demonstrated by our results or those of the most recent literature. Definitive conclusions concerning the role and safety of diagnostic hysteroscopy and the prognostic significance of different surgical procedures in EC patients can be drawn only from large prospective randomized trials.

## ETHICS STATEMENT

The Medical Ethics Committee of Tongji Hospital Affiliated to Tongji Medical College of Huazhong University of Science and Technology approved the study (No: TJ‐IRB20210737). All study participants provided informed written consent. The study was conducted ethically in accordance with the Helsinki World Medical Association Declaration.

## AUTHOR CONTRIBUTIONS

**Haiying Sun:** Data curation (equal); investigation (equal); resources (lead); writing – original draft (equal); writing – review and editing (equal). **Long Zhang:** Data curation (equal); formal analysis (equal); investigation (equal). **Peiying Fu:** Formal analysis (equal); investigation (equal); methodology (equal). **Ronghua Liu:** Conceptualization (lead); data curation (equal); project administration (lead).

## FUNDING INFORMATION

This work was supported by the National Natural Science Foundation of China (81572563).

## CONFLICT OF INTEREST STATEMENT

All authors declare that there are no conflicts of interest.

## Data Availability

The data used and analyzed during the study will be made available upon reasonable request made through the corresponding author.
